# Reanalysis of Chinese *Treponema pallidum* samples: all Chinese samples cluster with SS14-like group of syphilis-causing treponemes

**DOI:** 10.1186/s13104-017-3106-7

**Published:** 2018-01-11

**Authors:** Michal Strouhal, Jan Oppelt, Lenka Mikalová, Natasha Arora, Kay Nieselt, Fernando González-Candelas, David Šmajs

**Affiliations:** 1 Department of Biology, Faculty of Medicine, Masaryk University, Kamenice 5, Building A6, 625 00 Brno, Czech Republic; 2 CEITEC-Central European Institute of Technology, Masaryk University, Brno, Czech Republic; 3 National Centre for Biomolecular Research, Masaryk University, Brno, Czech Republic; 4 Zurich Institute of Forensic Medicine, University of Zurich, Zurich, Switzerland; 5 Center for Bioinformatics, University of Tübingen, Tübingen, Germany; 6 Unidad Mixta Infección y Salud Pública FISABIO, Universidad de Valencia, Valencia, Spain; 7 Institute for Integrative Systems Biology (I2SysBio), Universidad de Valencia-CSIC, Valencia, Spain; 8 CIBER Epidemiologia y Salud Pública (CIBERESP), Valencia, Spain

**Keywords:** *Treponema pallidum*, Syphilis, Genome sequencing, Phylogenetic analysis, Single nucleotide variant

## Abstract

**Objective:**

*Treponema pallidum* subsp. *pallidum* (TPA) is the causative agent of syphilis. Genetic analyses of TPA reference strains and human clinical isolates have revealed two genetically distinct groups of syphilis-causing treponemes, called Nichols-like and SS14-like groups. So far, no genetic intermediates, i.e. strains containing a mixed pattern of Nichols-like and SS14-like genomic sequences, have been identified. Recently, Sun et al. (Oncotarget 2016. 10.18632/oncotarget.10154) described a new “phylogenetic group” (called Lineage 2) among Chinese TPA strains. This lineage exhibited a “mosaic genomic structure” of Nichols-like and SS14-like lineages.

**Results:**

We reanalyzed the primary sequencing data (Project Number PRJNA305961) from the Sun et al. publication with respect to the molecular basis of Lineage 2. While Sun et al. based the analysis on several selected genomic single nucleotide variants (SNVs) and a subset of highly variable but phylogenetically poorly informative genes, which may confound the phylogenetic analysis, our reanalysis primarily focused on a complete set of whole genomic SNVs. Based on our reanalysis, only two separate TPA clusters were identified: one consisted of Nichols-like TPA strains, the other was formed by the SS14-like TPA strains, including all Chinese strains.

**Electronic supplementary material:**

The online version of this article (10.1186/s13104-017-3106-7) contains supplementary material, which is available to authorized users.

## Introduction

The bacterium *Treponema pallidum* subsp. *pallidum* (TPA) is the causative agent of syphilis. Other subspecies comprise *Treponema pallidum* subsp. *pertenue* (TPE) and *Treponema pallidum* subsp. *endemicum* (TEN), the causative agents of yaws and bejel, respectively. Since the pathogenic treponemes cannot be continuously cultivated under in vitro conditions, much of our understanding of these pathogens comes from accumulation of genetic and genomic data [1]. As previously shown by whole genome fingerprinting [2], analysis of several treponemal specific loci [3–5], whole genome sequence alignments of TPA reference strains [1, 6], and targeted whole genome sequencing of clinical isolates [7, 8], there is the evidence for two separate genetic subclusters within TPA treponemes, called Nichols-like and SS14-like, differing considerably at the DNA level (~ 400 nt differences).

Recently, Sun et al. [9] sequenced and analyzed eight TPA samples (propagated in rabbit testes) from syphilis-positive Chinese patients and compared them to the available treponemal genomes. Based on their results, a new “phylogenetic group” of TPA strains was identified and named Lineage 2 (Lineage 1 = Nichols-like, Lineage 3 = SS14-like). This Lineage 2 exhibited a “mosaic genomic structure” characterized by the insertion of Lineage 1-derived genes into the Lineage 3-derived genomic backbone. The authors also indicated that the analyzed Chinese TPA strains (Lineage 2) might be derived from recombination or lateral gene transfer events between Lineage 1 and Lineage 3. Until today, no genetic intermediates, i.e. strains containing a mixed pattern of Nichols-like and SS14-like genomic sequences, have been identified. Therefore, evidence for a new phylogenetic lineage would provide a new insight into the diversity and the phylogenetic relationships of TPA strains.

## Main text

### Methods

We reanalyzed the primary sequencing data from Sun et al. [9] with respect to the molecular basis of Lineage 2 using available SRA data (Illumina HiSeq 2500, 151 bp paired-end; Project Number PRJNA305961) and available TPA reference genomes. In contrast to Sun et al. [9], we used BWA MEM (instead of Bowtie2) and both Nichols and SS14 reference genome sequences for the genomic alignments and SNV analysis, supplemented with the de novo genome assembly analysis. Briefly, sequencing data were pre-processed with Trimmomatic. Sequencing reads were mapped to the reference genomes using BWA MEM. Resulting mappings were post-processed with Samtools to exclude low quality (MAPQ < 10), secondary, and not properly paired mappings. SNV for individual sequenced samples were called using FreeBayes. Hard-filters were applied to keep only high quality variants as recommended by FreeBayes authors; with a minimal depth of at least 5 (DP > 5) and variant call quality of at least 50 (QUAL > 50). Multiple whole genome alignment SNVs were called using NUCmer. Results were used in the phylogenetic analysis and processed with a custom R script. Only SNV detected in all analyzed samples were used in the analysis. For more details on the data collection and analysis, see Additional file 1.

### Results

In our analysis, we mapped 1.65–35.61% of all input read pairs to either the SS14 or the Nichols reference genome (see Additional file 2), with average coverage depth ranging between 58 and 1184× for both reference genomes (see Additional file 3). The remaining read pairs mapped primarily to the rabbit reference genome (57.01–89.80%; see Additional file 2). We achieved 99.19–99.35% and 98.87–99.08% genome coverage in the SS14 and Nichols genome alignments, respectively (see Additional file 3). The genomic regions which cannot be covered by uniquely mapped reads were located mainly in paralogous *tpr* genes (*C*, *D*, *E*, *F*, *G*, *I*, *J*, and *K*), RNA operons, and genes containing repetitive sequences, i.e. *tp0433* (*arp*) and *tp0470* (see Additional file 4). Overall mapping and genome coverage statistics calculated for the individual Chinese strains are summarized in additional files (see Additional files 2, 3 and 4).

**Fig. 1:**
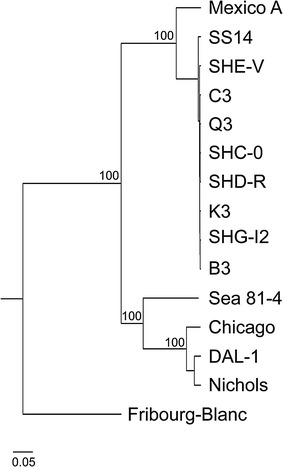
The phylogenetic tree of *Treponema* genomes—reposition of Chinese samples. The phylogenetic tree was constructed using hierarchical clustering based on multiscale bootstrap resampling. The number of bootstrap replications was set to 1000. The bar scale represents the number of substitutions per site. The analysis involved 14 TPA genome sequences including eight derived from the Chinese samples: SHC-0, SHD-R, SHE-V, SHG-I2, B3, C3, K3, and Q3. The *T. pallidum* subsp. *pertenue* Fribourg-Blanc genome sequence was used as an outgroup. There was a total of 2444 SNV positions in the final dataset (see Additional file 9). Two separate clusters supported by a bootstrap confidence level greater than 95% were identified: one cluster consisted of Nichols-like TPA strains (TPA Lineage 1), the other consisted of SS14-like TPA strains including all tested Chinese strains (TPA Lineage 3). A total of 1,130,357 nucleotide positions were aligned in the final dataset

Based on the multiple whole genome alignment of 14 TPA genome sequences, the number of identified SNVs obtained from the sequencing data for the individual Chinese samples ranged between 14 and 19 when compared to the SS14 reference genome and 282–327 when compared to the Nichols reference genome (see Additional file 5). Moreover, additional detailed variant calling analysis of the sequencing data of individual Chinese samples (using FreeBayes) was performed and showed similar results (data not shown). Two separate branches, supported by a bootstrap support greater than 95%, were identified: one consisted of Nichols, DAL-1, Chicago, and Sea 81-4 strains sharing the same phylogenetic cluster (Lineage 1), the other was formed by the SS14-like TPA strains, including all the Chinese strains (Lineage 3). The clustering of the Chinese samples within Lineage 3 is shown in the Fig. 1. A clustering of Chinese strains with genome sequences of clinical TPA isolates from different countries is shown in a supplementary material in Arora et al. (original Supplementary Figure 6) [7].

In addition, we identified the *tprD2* allele-specific sequence [10] among the sequencing reads from the Chinese SRA data in all samples (see Additional file 6). While the Nichols reference genome harbors identical copies of *tprC* and *tprD* genes, the SS14 reference genome carries the *tprD2* allele which is not identical to the *tprC* gene and differs from the *tprD* allele by more than 300 nucleotides. The sequence alignment of these alleles comprising the most variable region is shown in Additional file 7.

Moreover, all Chinese samples were found to contain indels identical to those identified in the SS14 genome (see Additional file 6) when compared to the Nichols-like TPA strains.

The analysis of two loci, *tp0136* and *tp0548*, potentially differentiating Nichols-like and SS14-like groups of TPA strains and isolates [11, 12], revealed that these genes were identical to the corresponding SS14 orthologous genes (see Additional file 8). In addition, the analysis of selected genes differentiating Nichols and SS14 reference strains at three or more nucleotide positions (*tp0131*, *tp0136*, *tp0179*, *tp0304*, *tp0346*, *tp0462*, *tp0488*, *tp0515*, *tp0548*, *tp0558*, and *tp1031*), showed them to be identical or nearly identical to the SS14 strain (data not shown).

### Discussion

The identification of two genetically distinct TPA lineages has been described by earlier genetic studies [5, 6] and these findings have been supported by recent whole genome sequencing studies [7, 8]. In Arora et al. [7], the phylogenetic analysis of 28 clinical isolates from different countries showed a clear separation of TPA isolates into two clusters, SS14-like and Nichols-like, although the Nichols-like cluster revealed greater variability. Moreover, Pinto et al. [8] described three clades—SS14-like (clade I), Nichols-like (clade II) and clade III (represented by only a single genome of the TPA Sea 81-4 strain [13]), it remains to be clarified whether this putative clade III will be supported by additional strains in the future. However, the Sea 81-4 strain shares the same phylogenetic cluster as the Nichols-like TPA strains (Fig. 1). Until now, no genetic intermediates having a mosaic structure of Nichols-like and SS14-like nucleotide sequences within TPA strains have been identified.

Sun et al. [9] described a new Lineage 2 of TPA based on a phylogenetic analysis of several genomic SNVs and sequences of *tpr* genes, presenting the Chinese strains as SS14-like strains containing recombined sequences originating from Nichols-like strains. This mosaic structure of Lineage 2 was characterized by the insertion of Lineage 1-derived genes (in particular *tprC*, *D*, *G*, and *J* genes) into the Lineage 3-derived genomic backbone.

There are, however, several issues in the presented analyses of Sun et al. [9]. Sun et al. reported the achievement of 99.99% of the genome coverage for all samples using TPA Nichols reference genome (original Table 1 in Sun et al. [9]). However, TPA genomes have several repetitive regions (representing ~ 1% of the genome length) which cannot be covered by uniquely mapped reads. These regions comprise mainly *tpr* genes and RNA operons. Long-read sequencing, such as Pacific Biosciences, Oxford Nanopore or even Roche/454, could help to sequence repeat-containing and paralogous regions. However, this sequencing was not performed by Sun et al. [9]. The Bowtie2 settings used by the authors without proper post-processing caused mappings of treponemal reads to wrong genomic locations as well as mapping of the host genome (rabbit) reads to the reference genome. The used read mapping stringency together with the use of inappropriate reference sequence (TPA Nichols) resulted in false chimeric sequences, designated as “Lineage 2”. Inappropriate consensus assembly conditions, combined with the absence of de novo assembly, resulted also in the overlooking of the presence of *tprD2* allele-specific sequences in the raw data and filtering out of these *tprD2* allele-specific sequences during assembly. Unlike the SS14 genome containing *tprC* and *tprD2* alleles, the Nichols reference genome harbors identical alleles in the *tprC* and *tprD* genes.

Sun et al. ([9], original Figure 4A) used alignment settings and phylogenetic trees of *tprC/D* loci to draw evolutionary inferences. The use of *tpr* genes alone to disentangle evolutionary relationships is problematic since these genes are likely subject to intra-strain genomic recombination events [3, 14–16] as well as selection pressures, which may confound phylogenetic analyses.

To date, all clinical isolates typed using *tp0136* and *tp0548* genes, routinely used in sequencing-based molecular typing scheme of syphilis-causing strains, grouped with either Nichols-like or SS14-like TPA groups [11, 12, 17, 18]. Moreover, more widely used enhanced CDC typing scheme sequencing an 83 nt-long fragment of the *tp0548* gene [19], showed that 94.5% of 1974 characterized clinical isolates from different countries belong to the SS14-like group [17], which is consistent with the findings related to a recent spread of an epidemic cluster [7]. For all the Chinese strains, *tp0136* and *tp0548* genes together with 11 other loci (differentiating Nichols and SS14 reference strains at three or more nucleotide positions) were shown to be identical or nearly identical to the SS14-like TPA strains.

Our reanalysis was based on all whole genomic SNVs rather than a subset of several genomic SNVs and highly variable but phylogenetically poorly informative genes (i.e. *tpr* genes) that confounded the Sun et al. [9] analysis and resulted in misleading conclusions. Based on the whole genome SNV reanalysis, only two separate clusters were identified: one consisted of Nichols-like TPA strains, the other was formed by the SS14-like TPA strains, including all the Chinese strains. Our data clearly showed that all Chinese strains clustered within SS14-like TPA strains.

## Limitations

- Only available SRA data deposited in the NCBI SRA database (Project Number PRJNA305961) were reanalysed.
- Only ~ 99% of genome coverage can be achieved for all TPA Chinese strains due to several repetitive regions which cannot be covered by uniquely mapped reads.
- Paralogous and repetitive genomic regions comprising *tpr* genes were excluded during the processing of the sequencing data, therefore mosaic structure identified in *tpr* genes by Sun et al. [9] cannot be proved.

## Additional files

**Additional file 1.** Data analysis and methods used in the reanalysis of Chinese *Treponema pallidum* samples.

**Additional file 2.** Mapping statistics of input read pairs mapped to the reference genomes. Sequencing reads derived from the Chinese strain SRA data were mapped to the *Treponema pallidum* subsp. *pallidum* (TPA) SS14 and Nichols reference genomes [6] and to the rabbit genome (Statistics was calculated from post-processed mappings; repetitive and homologous sequences and PCR duplicated reads were excluded from the statistics).

**Additional file 3.** Genome coverage statistics for individual Chinese strains. Sequencing reads derived from the Chinese strains SRA data were mapped to the TPA SS14 and Nichols reference genomes [6]. The number of bases with a coverage depth of 1 or more, number of bases with more than 10× coverage depth and average/median coverage depth are shown. Statistics were calculated from previously post-processed mappings; repetitive and homologous regions and PCR duplicated reads were excluded from statistical analysis.

**Additional file 4.** A Genome coverage statistics—number of non-covered bases for individual Chinese strains. SS14 genome (CP004011.1) [6] was used as a reference for mapping; coordinates according to the CP004011.1. The list includes all positions without any coverage by mapped reads. B Genome covearge statistics—number of zero covered bases for individual Chinese strains. Nichols genome (CP004010.2) [6] was used as a reference for mapping; coordinates according to the CP004010.2. The list includes all positions without any coverage by mapped reads.

**Additional file 5.** Number of SNVs from whole genome alignments produced by NUCmer. Only SNVs detected in all analyzed genomes (i.e., positions with the “N” base in any of the compared genomes were not considered) were used in the analysis. Genes *tp0433* (*arp*), *tp0470,* and *tp0897* (*tprK*) were excluded from analyses. Chinese strains are shown in bold.

**Additional file 6.** Analysis of indels (deletions/insertions) between SS14-like and Nichols-like TPA strains. The Nichols genome (CP004010.2) [6] was used as a reference for the comparison of TPA strains.

**Additional file 7.** Alignment of *tprD*/*tprD2* alleles. *tprD* and *tprD2* alleles were downloaded from the NCBI GenBank database for each reference Nichols and SS14 TPA strain, CP004010.2 and CP004011.1 [6], respectively. While the Nichols reference genome harbors identical copies of *tprC* and *tprD* genes, the SS14 reference genome carries the *tprD2* allele, which is not identical to the *tprC* gene and differs from the *tprD* allele by roughly 320 nucleotides. As shown in the alignment, we were able to identify the *tprD2* allele (in positions 800–1791 according to the SS14 *tprD2* allele) among the sequencing reads from the Chinese SRA data. The alignment was performed using SeqMan software (DNASTAR, Madison, WI, USA).

**Additional file 8.** The phylogenetic trees of the *tp0136* and *tp0548* genes. The phylogenetic trees were constructed using the Maximum Likelihood method based on the Tamura–Nei model. The bar scale represents the number of substitutions per site. The analysis involved 14 TPA nucleotide sequences including eight derived from the Chinese samples: SHC-0, SHD-R, SHE-V, SHG-I2, B3, C3, K3, and Q3. The *T. pallidum* subsp. *pertenue* Fribourg-Blanc sequence [1] was used as an outgroup. There were totals of 1547 and 1317 positions in the final dataset for *tp0136* and *tp0548* genes, respectively. For both genes, two separate clusters were identified: one cluster of Nichols-like TPA strains (TPA Lineage 1), and a second cluster of SS14-like TPA strains including all tested Chinese strains (TPA Lineage 3). Both clusters were supported by bootstrap values greater than 95%.

**Additional file 9.** Genomic SNVs used for phylogenetic analysis. List of SNVs used for construction of phylogenetic trees. Only SNVs detected in all analyzed genomes (i.e., positions with the “N” base in any of the compared genomes were not considered) were used in the analysis. Genes *tp0433* (*arp*), *tp0470*, and *tp0897* (*tprK*) were excluded from analyses. Altogether, 2444 unique SNV positions were identified when the TPE Fribourg-Blanc genome sequence was used as an outgroup. Coordinates (positions) and gene annotations according to the SS14 genome (CP04011.1); “NA” = not annotated (IGR, intergenic region); “.” = deletion.

## Abbreviations

TPA: *Treponema pallidum* subsp. *pallidum*
TPE: *Treponema pallidum* subsp. *pertenue*
TEN: *Treponema pallidum* subsp. *endemicum*
SRA: sequence read archive
SNV: single nucleotide variant
IGR: intergenic region
