# Supplementary material for: Reanalysis of Chinese Treponema pallidum samples: all Chinese samples cluster with SS14-like group of syphilis-causing treponemes
Source: BMC Res Notes. 2018 Jan 11;11:16. doi: 10.1186/s13104-017-3106-7 (PMC5765698; doi:10.1186/s13104-017-3106-7)
Supplement: Supplementary file 1 — Additional file 1. Data analysis and methods used in the reanalysis of Chinese Treponema pallidum samples. [file 13104_2017_3106_MOESM1_ESM.doc]

**Additional file 1. Data analysis and methods used in the reanalysis of Chinese *Treponema pallidum* samples.**

**Data collection**

Sequencing data derived from 8 Chinese TPA strains (SHC-0, SHD-R, SHE-V, SHG-I2, B3, C3, K3, and Q3) were downloaded from the NCBI SRA database (project number PRJNA305961) [1]. For comparison with other available TPA strains, the reference genome sequences for Nichols (CP004010.2) [2], DAL-1 (CP003115.1) [3], Chicago (CP001752.1) [4], SS14 (CP004011.1) [2], Mexico A (CP003064.1) [5], and Sea 81-4 (CP003679.1) [6] strains were downloaded from the NCBI Nucleotide database. TPE Fribourg-Blanc genome (CP003902.1) [7] was used as an outgroup in the analyses.

**Single nucleotide variants (SNV) determination and SNV data analysis**

The downloaded sequencing data were converted to the FASTQ format and split into paired-end sequences using a SRA Toolkit (2.5.7) [8]. The quality of the sequencing data was checked using FastQC (0.11.5) [9] and the presence of adapters was checked using minion and swan (Kraken toolkit, 13.100) [10]. Samples were pre-processed with Trimmomatic (0.36) [11] where “N” and very low quality bases (Phred < 3) were removed from both ends. Low quality bases were additionally removed with a sliding window of length 4 with an average quality of at least Phred 10. Sequencing reads shorter than 35 bp after pre-processing were removed.

Sequencing reads were mapped to the Nichols and SS14 reference genomes (CP004010.2 [2] and CP004011.1 [2], respectively) using BWA MEM (0.7.5a) [12]. Mappings were post-processed with Samtools (1.3) [13] to exclude low quality (MAPQ < 10), secondary, and not properly paired mappings. PCR deduplication and optical sequencing duplicates as well as indel realignment was performed using Picard tools (2.5.0) [14] and GATK (3.6) [15]. Genome coverage and coverage depth was calculated using Bedtools (2.17) [16]. SNV for individual sequenced samples were called using FreeBayes (9.9.2) [17]. Hard-filters were applied to keep only high quality variants as recommended by FreeBayes authors; with a minimal depth of at least 5 (DP > 5) and variant call quality of at least 50 (QUAL > 50). Due to the presence of repetitive sequences and/or intrastrain variable sites, genes *tp0433* (*arp*), *tp0470,* and *tp0897* (*tprK*) were excluded from the SNV analysis.

***De novo* genome assembly**

Raw sequencing reads were pre-processed in the same way as in the SNV discovery. All sequencing reads were then mapped to the rabbit reference genome (GCF_000003625.3; available from: https://www.ncbi.nlm.nih.gov/assembly/182491) to remove possible host genome contamination. Mapping was performed with the same settings as in the SNV discovery. Potential treponemal reads, i.e. sequencing reads that did not map to the rabbit genome and reads that mapped to the rabbit genome with mapping quality lower than 30 (MAPQ < 30) were used for *de novo* assembly, using SPAdes (3.9.0) [18] with set of k-mers ranging from 17–77 (in total, there were 16 different k-mer lengths). Sequencing read correction was performed before the assembly and it is included in the assembler by default. Estimated genome sizes of individual Chinese strains were calculated based on the selection of possibly connected contigs from SPAdes assembly, which was visualized with the Bandage software [19].

**Phylogenetic analysis of treponemal strains**

Consensus genomes for the Chinese strains were generated from alignment using Samtools pileup (0.1.16 and 0.1.19) [13] and compared to the other available TPA reference genomes. Multiple whole genome alignment SNVs were called using NUCmer (MUMmer package, 3.23) [20]. Results from NUCmer were used in the phylogenetic analysis and processed with a custom R script (3.3.1) [21] using Biostrings (2.38.4) [22], pvclust (2.0-0) [23], and ape (3.5) [24] packages. Only SNV detected in all analyzed samples (positions with the “N” base in any of the compared genomes were not considered) were used in the analysis. As with the SNV determination, genes containing highly variable or repetitive sequences (*tp0433*, *tp0470* and *tprK*) were excluded from the analysis.

*tp0136* and *tp0548* genes were downloaded from the NCBI GenBank annotation for each reference TPA genome. The orthologous sequences for these genes were extracted from the Chinese consensus genomes and aligned with *tp0136* and *tp0548* reference gene sequences using SeqMan software (DNASTAR, Madison, WI, USA). The construction of phylogenetic trees using these genes was carried out using MEGA7 [25], which utilized the Maximum Likelihood method based on the Tamura-Nei model [26].

**Supplementary information file references**

1. Sun J, Meng Z, Wu K, Liu B, Zhang S, Liu Y, et al. Tracing the origin of *Treponema pallidum* in China using next-generation sequencing. Oncotarget. 2016; doi:10.18632/oncotarget.10154.
2. Pětrošová H, Pospíšilová P, Strouhal M, Čejková D, Zobaníková M, Mikalová L, et al. Resequencing of *Treponema pallidum* ssp. *pallidum* strains Nichols and SS14: correction of sequencing errors resulted in increased separation of syphilis treponeme subclusters. PLoS One. 2013;8:e74319.
3. Zobaníková M, Mikolka P, Čejková D, Pospíšilová P, Chen L, Strouhal M, et al. Complete genome sequence of *Treponema pallidum* strain DAL-1. Stand Genomic Sci. 2012;7:12-21.
4. Giacani L, Jeffrey BM, Molini BJ, Le HT, Lukehart SA, Centurion-Lara A, et al. Complete genome sequence and annotation of the *Treponema pallidum* subsp. *pallidum* Chicago strain. J Bacteriol. 2010;192:2645-2646.
5. Pětrošová H, Zobaníková M, Čejková D, Mikalová L, Pospíšilová P, Strouhal M, et al. Whole genome sequence of *Treponema pallidum* ssp. *pallidum*, strain Mexico A, suggests recombination between yaws and syphilis strains. PLoS Negl Trop Dis. 2012;6:e1832.
6. Giacani L, Iverson-Cabral SL, King JC, Molini BJ, Lukehart SA, Centurion-Lara A. Complete genome sequence of the *Treponema pallidum* subsp. *pallidum* Sea81-4 strain. Genome Announc. 2014;2:e00333-14.
7. Zobaníková M, Strouhal M, Mikalová L, Čejková D, Ambrožová L, Pospíšilová P, et al. Whole genome sequence of the *Treponema* Fribourg-Blanc: unspecified simian isolate is highly similar to the yaws subspecies. PLoS Negl Trop Dis. 2013;7:e2172.
8. SRA Toolkit Development Team. 2016. Available from: <https://trace.ncbi.nlm.nih.gov/Traces/sra/sra.cgi?view=software>.
9. Andrews S. FastQC: a quality kontrol tool for high throughput sequence data. 2010. Available from: <http://www.bioinformatics.babraham.ac.uk/projects/fastqc>.
10. Davis MP, van Dongen S, Abreu-Goodger C, Bartonicek N, Enright AJ. Kraken: a set of tools for quality control and analysis of high-throughput sequence data. Methods. 2013;63:41-49.
11. Bolger AM, Lohse M, Usadel B. Trimmomatic: a flexible trimmer for Illumina sequence data. Bioinformatics. 2014;30:2114-2120.
12. Li H. Aligning sequence reads, clone sequences and assembly contigs with BWA-MEM. 2013. Available from: arXiv:1303.3997v2 [q-bio.GN].
13. Li H, Handsaker B, Wysoker A, Fennell T, Ruan J, Homer N, et al. 1000 Genome Project Data Processing Subgroup. The Sequence Alignment/Map format and SAMtools. Bioinformatics. 2009;25:2078-2079.
14. Pikard. 2016. Available from: [http://picard.sourceforge.net](http://picard.sourceforge.net/).
15. McKenna A, Hanna M, Banks E, Sivachenko A, Cibulskis K, Kernytsky A, et al. The Genome Analysis Toolkit: a MapReduce framework for analyzing next-generation DNA sequencing data. Genome Res. 2010;20: 1297-1303.
16. Quinlan AR, Hall IM. BEDTools: a flexible suite of utilities for comparing genomic features. Bioinformatics. 2010;26:841-842.
17. Garrison E., Marth G. Haplotype-based variant detection from short-read sequencing. 2012. Available from: arXiv:1207.3907 [q-bio.GN].
18. Nurk S, Bankevich A, Antipov D, Gurevich AA, Korobeynikov A, Lapidus A, et al. Assembling single-cell genomes and mini-metagenomes from chimeric MDA products. J Comput Biol. 2013;20:714-737.
19. [Wick RR, Schultz MB, Zobel J, Holt KE. Bandage: interactive visualisation of *de novo* genome assemblies. Bioinformatics. 2015;31:3350-3352.](http://bioinformatics.oxfordjournals.org/content/31/20/3350)
20. Kurtz S, Phillippy A, Delcher AL, Smoot M, Shumway M, Antonescu C, et al. Versatile and open software for comparing large genomes. Genome Biol. 2004;5:R12.
21. R Development Core Team. R: A Language and Environment for Statistical Computing. Vienna, Austria: the R Foundation for Statistical Computing. 2016; ISBN: 3-900051-07-0. Available from: <http://www.R-project.org/>.
22. Pages H, Aboyoun P, Gentleman R, DebRoy S. Biostrings: String objects representing biological sequences, and matching algorithms. 2016. R package version 2.38.4.
23. Suzuki R, Shimodaira H. Pvclust: an R package for assessing the uncertainty in hierarchical clustering. Bioinformatics. 2006;22:1540-1542.
24. Paradis E, Claude J, Strimmer K. APE: Analyses of Phylogenetics and Evolution in R language. Bioinformatics. 2004;20:289-290.
25. Kumar S, Stecher G, Tamura K. MEGA7: Molecular Evolutionary Genetics Analysis version 7.0 for bigger datasets. Mol Biol Evol. 2015;33(7):1870-1874.
26. Tamura K, Nei M. Estimation of the number of nucleotide substitutions in the control region of mitochondrial DNA in humans and chimpanzees. Mol Biol Evol. 1993;10:512-526.
