# Supplementary material for: Reanalysis of Chinese Treponema pallidum samples: all Chinese samples cluster with SS14-like group of syphilis-causing treponemes
Source: BMC Res Notes. 2018 Jan 11;11:16. doi: 10.1186/s13104-017-3106-7 (PMC5765698; doi:10.1186/s13104-017-3106-7)
Supplement: Supplementary file 2 — Additional file 2. Mapping statistics of input read pairs mapped to the reference genomes. Sequencing reads derived from the Chinese strain SRA data were mapped to the Treponema pallidum subsp. pallidum (TPA) SS14 and Nichols reference genomes [6] and to the rabbit genome (Statistics was calculated from post-processed mappings; repetitive and homologous sequences and PCR duplicated reads were excluded from the statistics). [file 13104_2017_3106_MOESM2_ESM.doc]

**Additional file 2. Mapping statistics of input read pairs mapped to the reference genomes.** Sequencing reads derived from the Chinese strain SRA data were mapped to the *Treponema pallidum* subsp. *pallidum* (TPA) SS14 and Nichols reference genomes and to the rabbit genome. (Statistics was calculated from post-processed mappings; repetitive and homologous sequences and PCR duplicated reads were excluded from the statistics.)

| **SRA Run** | **Sample** | **Original Input Read Pairs** | | **Quality Pre-processed Input Read Pairs** | | **Read pairs mapped to TPA SS14^a^** | | **Read pairs mapped to TPA Nichols^a^** | | **Read pairs mapped to rabbit^b^** | |
| --- | --- | --- | --- | --- | --- | --- | --- | --- | --- | --- | --- |
|  |  | Number of read pairs | % | Number of read pairs | % | Number of read pairs | % | Number of read pairs | % | Number of read pairs | % |
| SRR2996724 | SHC-0 | 20208147 | 100 | 18837183 | 93.22 | 4035354 | 21.42 | 4021896 | 21.35 | 13275018 | 70.47 |
| SRR2996725 | SHD-R | 14161953 | 100 | 13185024 | 93.10 | 4695775 | 35.61 | 4680114 | 35.50 | 7516316 | 57.01 |
| SRR2996726 | SHE-V | 13176446 | 100 | 12212541 | 92.68 | 1021764 | 8.37 | 1018532 | 8.34 | 10142819 | 83.05 |
| SRR2996727 | SHG-I2 | 11570830 | 100 | 10493927 | 90.69 | 484421 | 4.62 | 482907 | 4.60 | 9039474 | 86.14 |
| SRR2996728 | B3 | 13263670 | 100 | 13198603 | 99.51 | 218964 | 1.66 | 218282 | 1.65 | 11852624 | 89.80 |
| SRR2996729 | C3 | 13373048 | 100 | 13313706 | 99.56 | 1123688 | 8.44 | 1119946 | 8.41 | 11115550 | 83.49 |
| SRR2996730 | K3 | 12752433 | 100 | 12666301 | 99.32 | 1747631 | 13.80 | 1741818 | 13.75 | 9929639 | 78.39 |
| SRR2996732 | Q3 | 12409555 | 100 | 12350898 | 99.53 | 4109471 | 33.27 | 4095679 | 33.16 | 7374045 | 59.70 |

^a^SS14 (CP004011.1) and Nichols (CP004010.2) reference genomes

^b^GCF_000003625.3; available from: <https://www.ncbi.nlm.nih.gov/assembly/182491>
