# Supplementary material for: Reanalysis of Chinese Treponema pallidum samples: all Chinese samples cluster with SS14-like group of syphilis-causing treponemes
Source: BMC Res Notes. 2018 Jan 11;11:16. doi: 10.1186/s13104-017-3106-7 (PMC5765698; doi:10.1186/s13104-017-3106-7)
Supplement: Supplementary file 3 — Additional file 3. Genome coverage statistics for individual Chinese strains. Sequencing reads derived from the Chinese strains SRA data were mapped to the TPA SS14 and Nichols reference genomes [6]. The number of bases with a coverage depth of 1 or more, number of bases with more than 10× coverage depth and average/median coverage depth are shown. Statistics were calculated from previously post-processed mappings; repetitive and homologous regions and PCR duplicated reads were excluded from statistical analysis. [file 13104_2017_3106_MOESM3_ESM.doc]

**Additional file 3. Genome coverage statistics for individual Chinese strains.** Sequencing reads derived from the Chinese strains SRA data were mapped to the TPA SS14 and Nichols reference genomes. The number of bases with a coverage depth of 1 or more, number of bases with more than 10x coverage depth and average/median coverage depth are shown. Statistics were calculated from previously post-processed mappings; repetitive and homologous regions and PCR duplicated reads were excluded from statistical analysis.

| **SRA Run** | **Sample** | **Non-zero covered bases^b^** | **Non-zero covered bases %** | **>10 covered bases^c^** | **>10 covered bases %** | **Average coverage^d^** | **Median coverage^d^** |
| --- | --- | --- | --- | --- | --- | --- | --- |
| **SS14 genome (1139569 nt)^a^** | |  |  |  |  |  |  |
| SRR2996724 | SHC-0 | 1131648 | 99.30 | 1130499 | 99.20 | 1017.84 | 1020 |
| SRR2996725 | SHD-R | 1131742 | 99.31 | 1130847 | 99.23 | 1184.16 | 1182 |
| SRR2996726 | SHE-V | 1131767 | 99.32 | 1129969 | 99.16 | 257.12 | 256 |
| SRR2996727 | SHG-I2 | 1132187 | 99.35 | 1129950 | 99.16 | 121.34 | 121 |
| SRR2996728 | B3 | 1130357 | 99.19 | 1126570 | 98.86 | 58.03 | 57 |
| SRR2996729 | C3 | 1130963 | 99.24 | 1129292 | 99.10 | 298.11 | 295 |
| SRR2996730 | K3 | 1131203 | 99.27 | 1129802 | 99.14 | 462.04 | 460 |
| SRR2996732 | Q3 | 1131468 | 99.29 | 1130253 | 99.18 | 1090.15 | 1082 |
| **Nichols genome (1139633 nt)^a^** | |  |  |  |  |  |  |
| SRR2996724 | SHC-0 | 1128434 | 99.02 | 1126890 | 98.88 | 1017.32 | 1020 |
| SRR2996725 | SHD-R | 1128727 | 99.04 | 1127530 | 98.94 | 1183.32 | 1181 |
| SRR2996726 | SHE-V | 1128374 | 99.01 | 1126190 | 98.82 | 257.07 | 256 |
| SRR2996727 | SHG-I2 | 1129154 | 99.08 | 1126278 | 98.83 | 121.28 | 121 |
| SRR2996728 | B3 | 1126727 | 98.87 | 1121789 | 98.43 | 58.04 | 57 |
| SRR2996729 | C3 | 1127523 | 98.94 | 1125189 | 98.73 | 298.01 | 295 |
| SRR2996730 | K3 | 1128101 | 98.99 | 1126105 | 98.81 | 461.75 | 460 |
| SRR2996732 | Q3 | 1128359 | 99.01 | 1126751 | 98.87 | 1089.43 | 1082 |

^a^SS14 (CP004011.1) and Nichols (CP004010.2) reference genomes

^b^number of bases with coverage depth 1 and more

^c^number of bases with more than 10x coverage depth

^d^average and median coverage was calculated from non-zero covered bases (i.e., zero covered bases were excluded from the analysis)
