# Supplementary material for: Reanalysis of Chinese Treponema pallidum samples: all Chinese samples cluster with SS14-like group of syphilis-causing treponemes
Source: BMC Res Notes. 2018 Jan 11;11:16. doi: 10.1186/s13104-017-3106-7 (PMC5765698; doi:10.1186/s13104-017-3106-7)
Supplement: Supplementary file 5 — Additional file 5. Number of SNVs from whole genome alignments produced by NUCmer. Only SNVs detected in all analyzed genomes (i.e., positions with the “N” base in any of the compared genomes were not considered) were used in the analysis. Genes tp0433 (arp), tp0470, and tp0897 (tprK) were excluded from analyses. Chinese strains are shown in bold. [file 13104_2017_3106_MOESM5_ESM.doc]

Additional file 5. Number of SNVs from whole genome alignments produced by NUCmer. Only SNVs detected in all analyzed genomes (i.e., positions with the “N” base in any of the compared genomes were not considered) were used in the analysis. Genes *tp0433* (*arp*), *tp0470,* and *tp0897* (*tprK*) were excluded from analyses. Chinese strains are shown in bold.

| **Comparison with SS14 genome (CP004011.1)** | | **Comparison with Nichols genome (CP004010.2)** | |
| --- | --- | --- | --- |
| **Number of SNVs** | **Compared genome** | **Number of SNVs** | **Compared genome** |
| 576 | Chicago_CP001752.1 | 30 | Chicago_CP001752.1 |
| 674 | DAL-1_CP003115.1 | 100 | DAL-1_CP003115.1 |
| 2106 | Fribourg-Blanc_CP0030902.1 | 2105 | Fribourg-Blanc_CP0030902.1 |
| 211 | Mexico A_CP003064.1 | 689 | Mexico A_CP003064.1 |
| 594 | Nichols_CP004010.2 | 0 | Nichols_CP004010.2 |
| 666 | Sea 81-4_CP003679.1 | 509 | Sea 81-4_CP003679.1 |
| 0 | SS14_CP004011.1 | 594 | SS14_CP004011.1 |
| **16** | **SHC-0_SRR2996724** | **327** | **SHC-0_SRR2996724** |
| **17** | **SHD-R_SRR2996725** | **326** | **SHD-R_SRR2996725** |
| **19** | **SHE-V_SRR2996726** | **317** | **SHE-V_SRR2996726** |
| **15** | **SHG-I2_SRR2996727** | **307** | **SHG-I2_SRR2996727** |
| **14** | **B3_SRR2996728** | **282** | **B3_SRR2996728** |
| **15** | **C3_SRR2996729** | **307** | **C3_SRR2996729** |
| **15** | **K3_SRR2996730** | **318** | **K3_SRR2996730** |
| **17** | **Q3_SRR2996732** | **320** | **Q3_SRR2996732** |
