# Supplementary material for: Reanalysis of Chinese Treponema pallidum samples: all Chinese samples cluster with SS14-like group of syphilis-causing treponemes
Source: BMC Res Notes. 2018 Jan 11;11:16. doi: 10.1186/s13104-017-3106-7 (PMC5765698; doi:10.1186/s13104-017-3106-7)
Supplement: Supplementary file 6 — Additional file 6. Analysis of indels (deletions/insertions) between SS14-like and Nichols-like TPA strains. The Nichols genome (CP004010.2) [6] was used as a reference for the comparison of TPA strains. [file 13104_2017_3106_MOESM6_ESM.doc]

**Additional file 6. Analysis of indels (deletions/insertions) between SS14-like and Nichols-like TPA strains.** The Nichols genome (CP004010.2) [1] was used as a reference for the comparison of TPA strains.

| **Indel coordinates^a^** | **Affected gene/IGR^b^** | **SS14-like strains^c^** | **Nichols-like strains^d^** | **Chinese strains^e^** |
| --- | --- | --- | --- | --- |
| 158339 - 158341 | *tp0136* | 3 nt deletion | TCA | 3 nt deletion |
| 158737 - 158742 | *tp0136* | 6 nt deletion | TGGCGG | 6 nt deletion |
| 159547 | IGR *tp0136* - *tp0138* | 1 nt deletion | T | 1 nt deletion |
| 320169 - 320171 | *tp0304* | 3 nt deletion | CTG | 3 nt deletion |
| 731907 - 731912 | *tp0668* | 6 nt deletion | ACAGCA | 6 nt deletion |
| 949048 - 949054 | *tp0868* | 7 nt deletion | AACGGCA | 7 nt deletion |
| between 149494 - 149495 | IGR *tp0126* - *tp0127* | 51 nt insertion^f^ | - | 51 nt insertion |
| between 150567 - 150568 | IGR *tp0127* - *tp0128* | 6 nt insertion CAGGGC | - | 6 nt insertion |
| between 587828 - 587829 | *tp0544* | 6 nt insertion TTCCTG | - | 6 nt insertion |
| between 593421 - 593422 | *tp0548* | 3 nt insertion TGG or TAG | - | 3 nt insertion |
| between 593732 - 593733 | *tp0548* | 9 nt insertion ACGGTATGA | - | 9 nt insertion |
| between 945695 - 945696 | *tp0865* | 3 nt insertion GTT | - | 3 nt insertion |
| 152314 - 154110 | *tp0131* | *tprD2* allele^g^ | *tprD* allele | *tprD2* allele |

^a^coordinates according to the Nichols strain (CP004010.2) [1]; genes *tp0433* and *tp0470* containing repetitive sequences, and the *tp0897* (*tprK*) gene containing variable regions and variable G/C homopolymeric regions were omitted from this analysis

^b^IGR - intergenic region

^c^SS14-like strains: SS14 (CP004011.1) [1], Mexico A (CP003064.1) [2], and 23 Portuguese strains (PT_SIF strains) obtained from Pinto *et al.* [3]

^d^Nichols-like strains: Nichols (CP004010.2) [1], DAL-1 (CP003115.1) [4], Chicago (CP001752.1) [5], and Sea 81-4 (CP003679.1) [6]

^e^Chinese strains: SHC-0, SHD-R, SHE-V, SHG-I2, B3, C3, K3, and Q3 [7]

^f^AAGAGCAGCAGCACCAGGAACTGCACCAGGGACCGCCCCAGGAACTGCACC

^g^*tprD2* allele differs from *tprD* allele in ≈ 320 nucleotides (see also Additional file 7)

References

1. Pětrošová H, Pospíšilová P, Strouhal M, Čejková D, Zobaníková M, Mikalová L, et al. Resequencing of *Treponema pallidum* ssp.*pallidum* strains Nichols and SS14: correction of sequencing errors resulted in increased separation of syphilis treponeme subclusters. PLoS One. 2013;8:e74319.
2. Pětrošová H, Zobaníková M, Čejková D, Mikalová L, Pospíšilová P, Strouhal M, et al. Whole genome sequence of *Treponema pallidum* ssp. *pallidum*, strain Mexico A, suggests recombination between yaws and syphilis strains. PLoS Negl Trop Dis. 2012;6:e1832.
3. Pinto M, Borges V, Antelo M, Pinheiro M, Nunes A, Azevedo J, et al. Genome-scale analysis of the non-cultivable *Treponema pallidum* revers extensit within-patient genetic variation. Nat Microbiol. 2016;2:16190.
4. Zobaníková M, Mikolka P, Čejková D, Pospíšilová P, Chen L, Strouhal M, et al. Complete genome sequence of *Treponema pallidum* strain DAL-1. Stand Genomic Sci. 2012;7:12-21.
5. Giacani L, Jeffrey BM, Molini BJ, Le HT, Lukehart SA, Centurion-Lara A, et al. Complete genome sequence and annotation of the *Treponema pallidum* subsp. *pallidum* Chicago strain. J Bacteriol. 2010;192:2645-2646.
6. Giacani L, Iverson-Cabral SL, King JC, Molini BJ, Lukehart SA, Centurion-Lara A. Complete genome sequence of the *Treponema pallidum* subsp. *pallidum* Sea81-4 strain. Genome Announc. 2014;2:e00333-14.
7. Sun J, Meng Z, Wu K, Liu B, Zhang S, Liu Y, et al. Tracing the origin of *Treponema pallidum* in China using next-generation sequencing. Oncotarget. 2016; doi:10.18632/oncotarget.10154.
