# Supplementary material for: Reanalysis of Chinese Treponema pallidum samples: all Chinese samples cluster with SS14-like group of syphilis-causing treponemes
Source: BMC Res Notes. 2018 Jan 11;11:16. doi: 10.1186/s13104-017-3106-7 (PMC5765698; doi:10.1186/s13104-017-3106-7)

**Additional file 7. Alignment of *tprD*/*tprD2* alleles.** *tprD* and *tprD2* alleles were downloaded from the NCBI GenBank database for each reference Nichols and SS14 TPA strain, CP004010.2 and CP004011.1, respectively. While the Nichols reference genome harbors identical copies of *tprC* and *tprD* genes, the SS14 reference genome carries the *tprD2* allele, which is not identical to the *tprC* gene and differs from the *tprD* allele by roughly 320 nucleotides. As shown in the alignment, we were able to identify the *tprD2* allele (in positions 800–1791 according to the SS14 *tprD2* allele) among the sequencing reads from the Chinese SRA data. The alignment was performed using SeqMan software (DNASTAR, Madison, WI, USA).


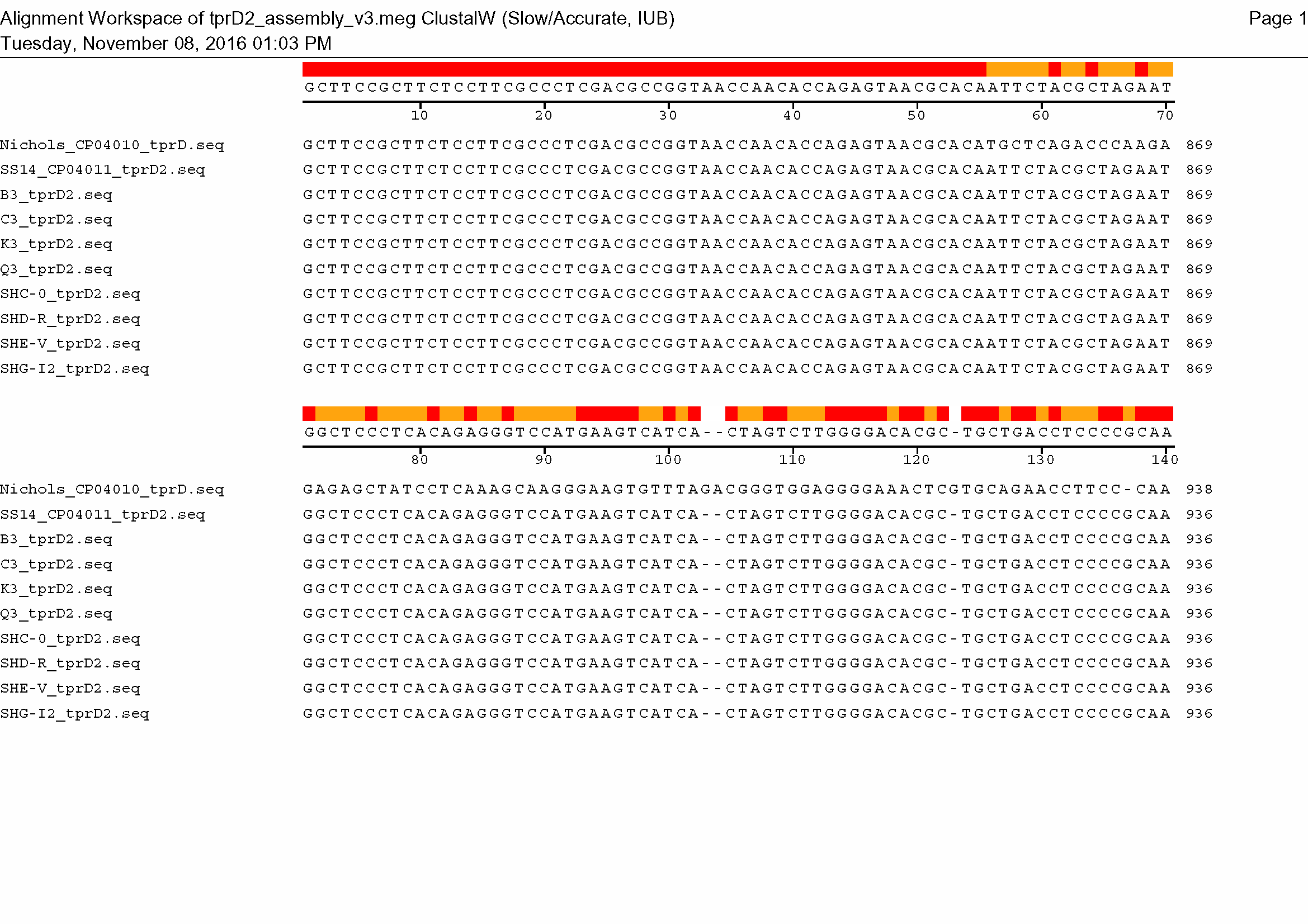


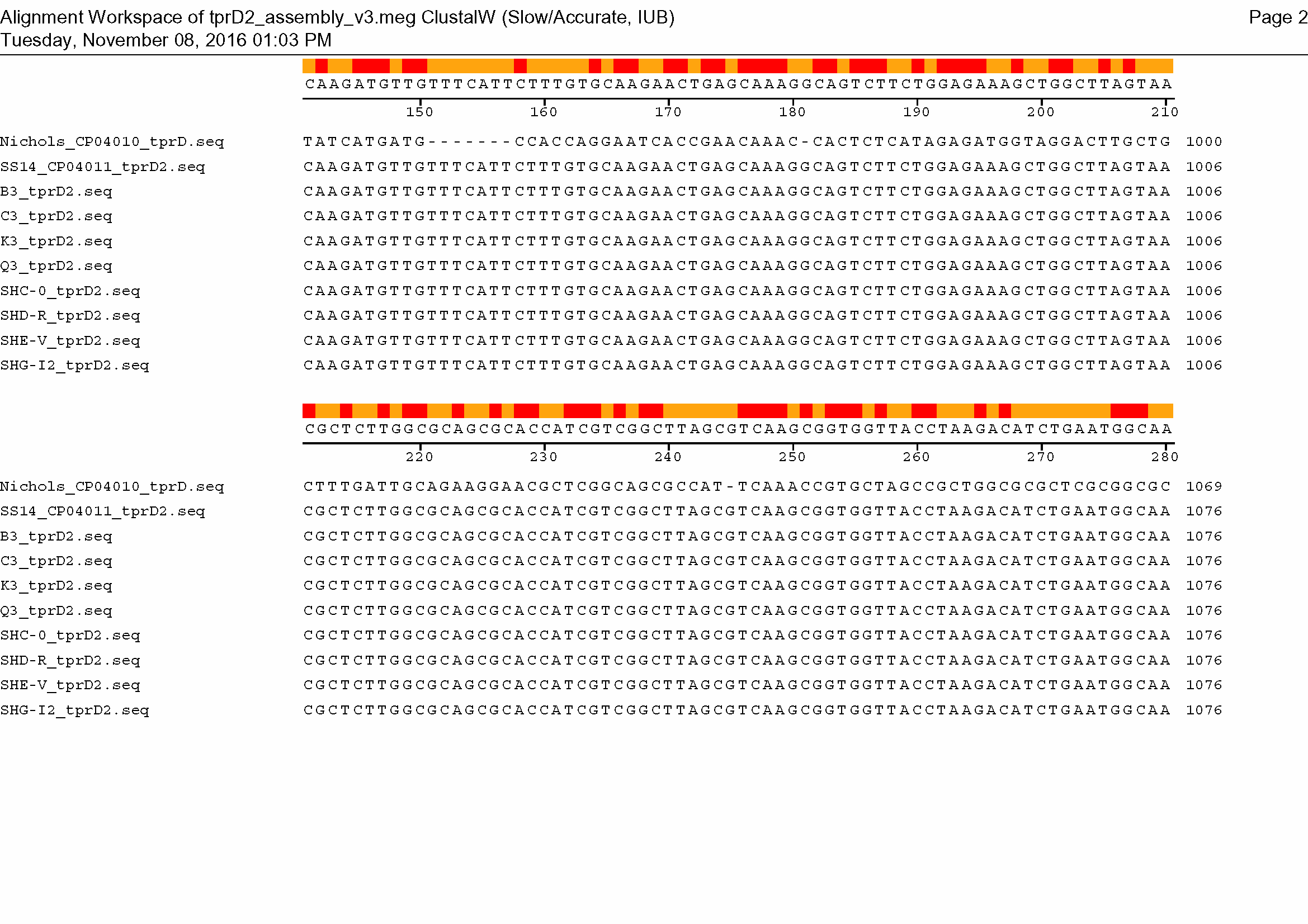


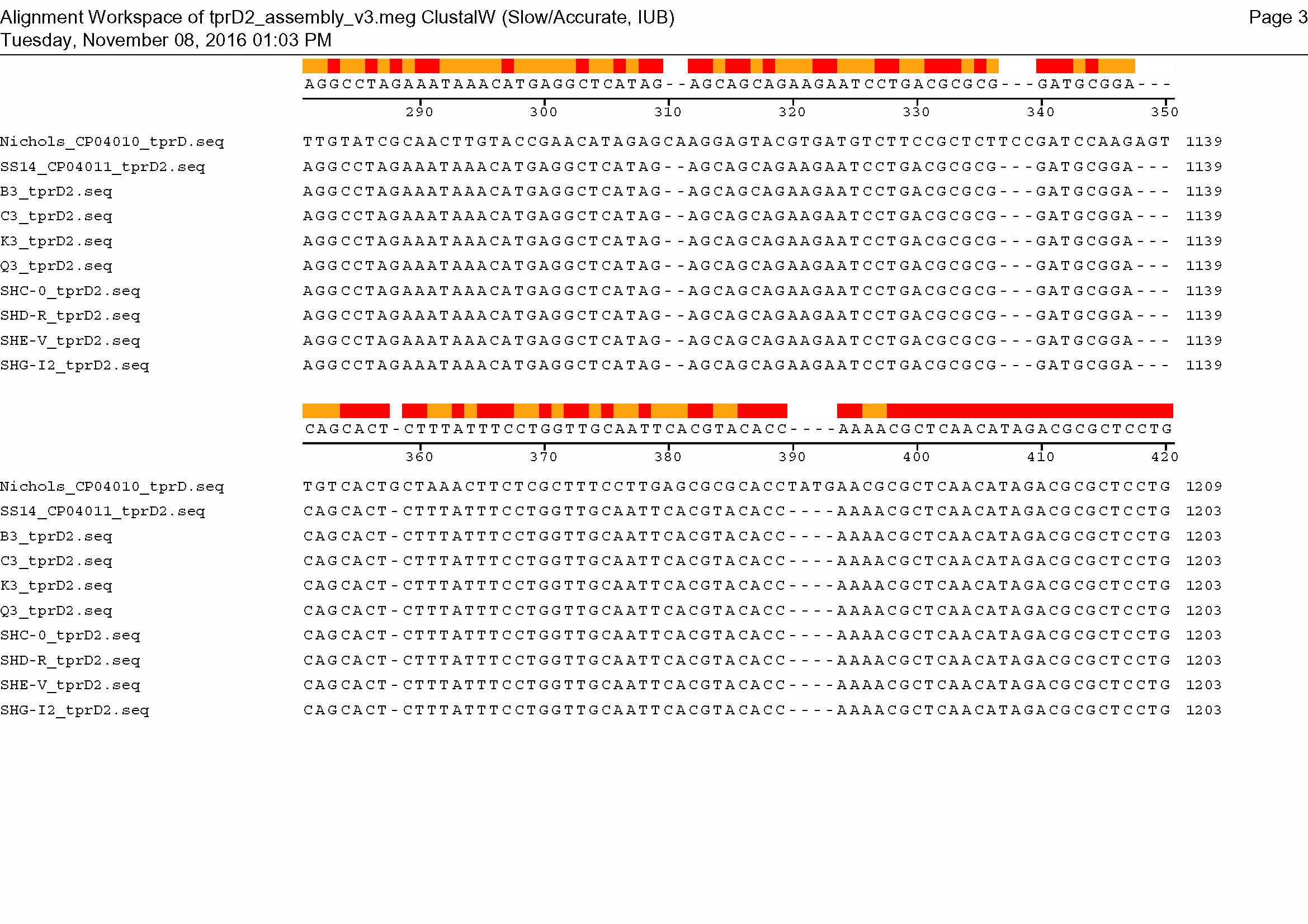


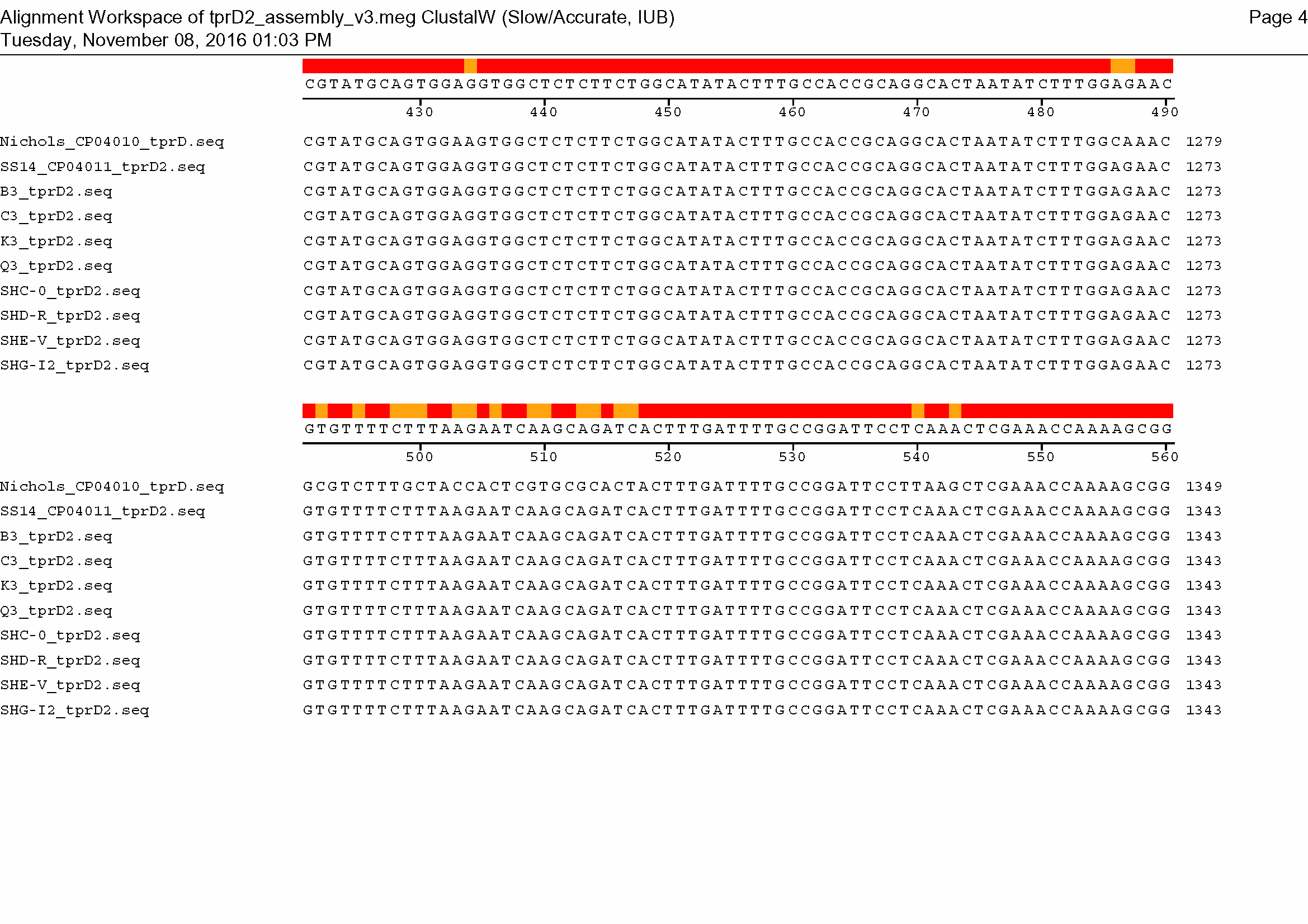

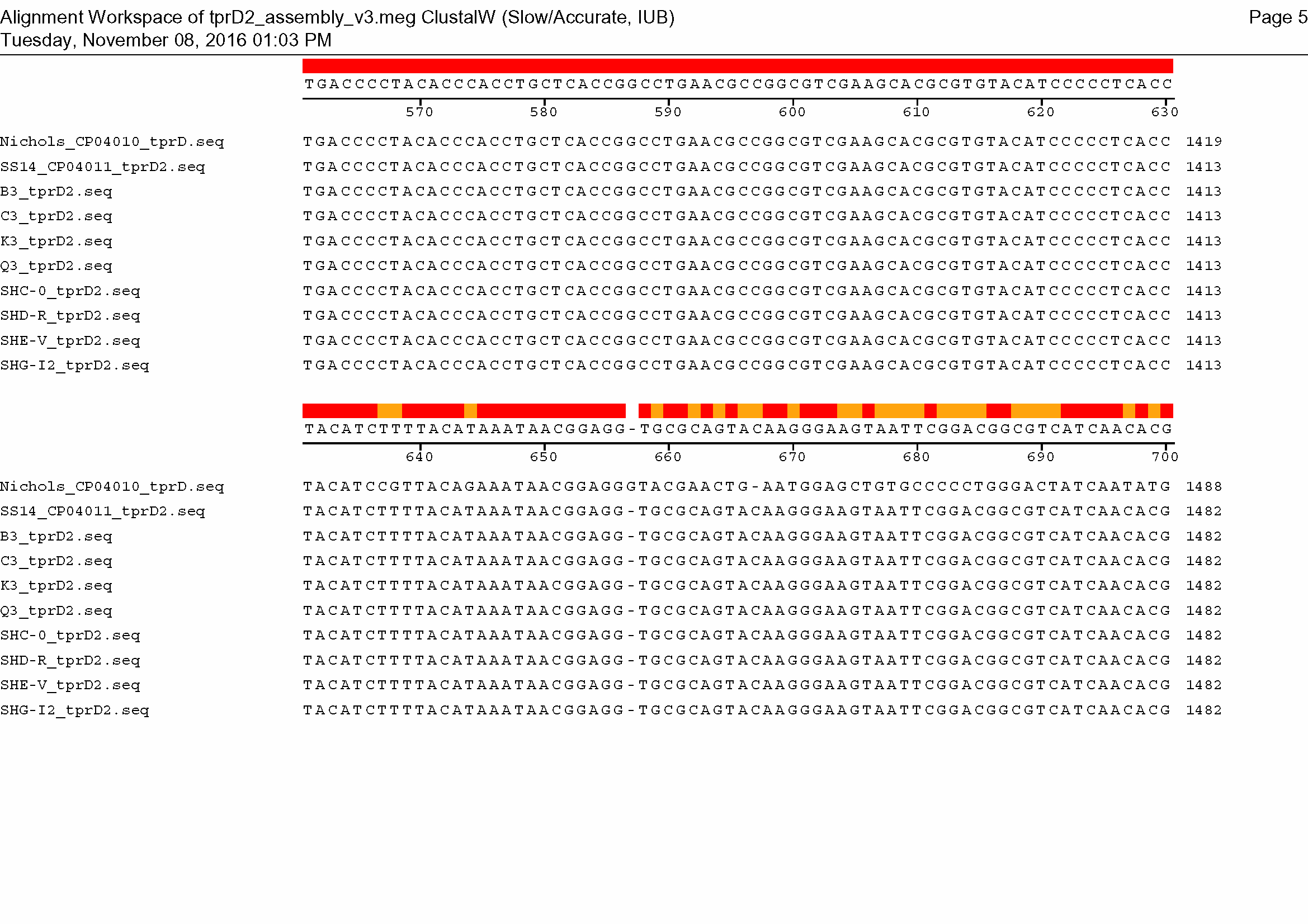


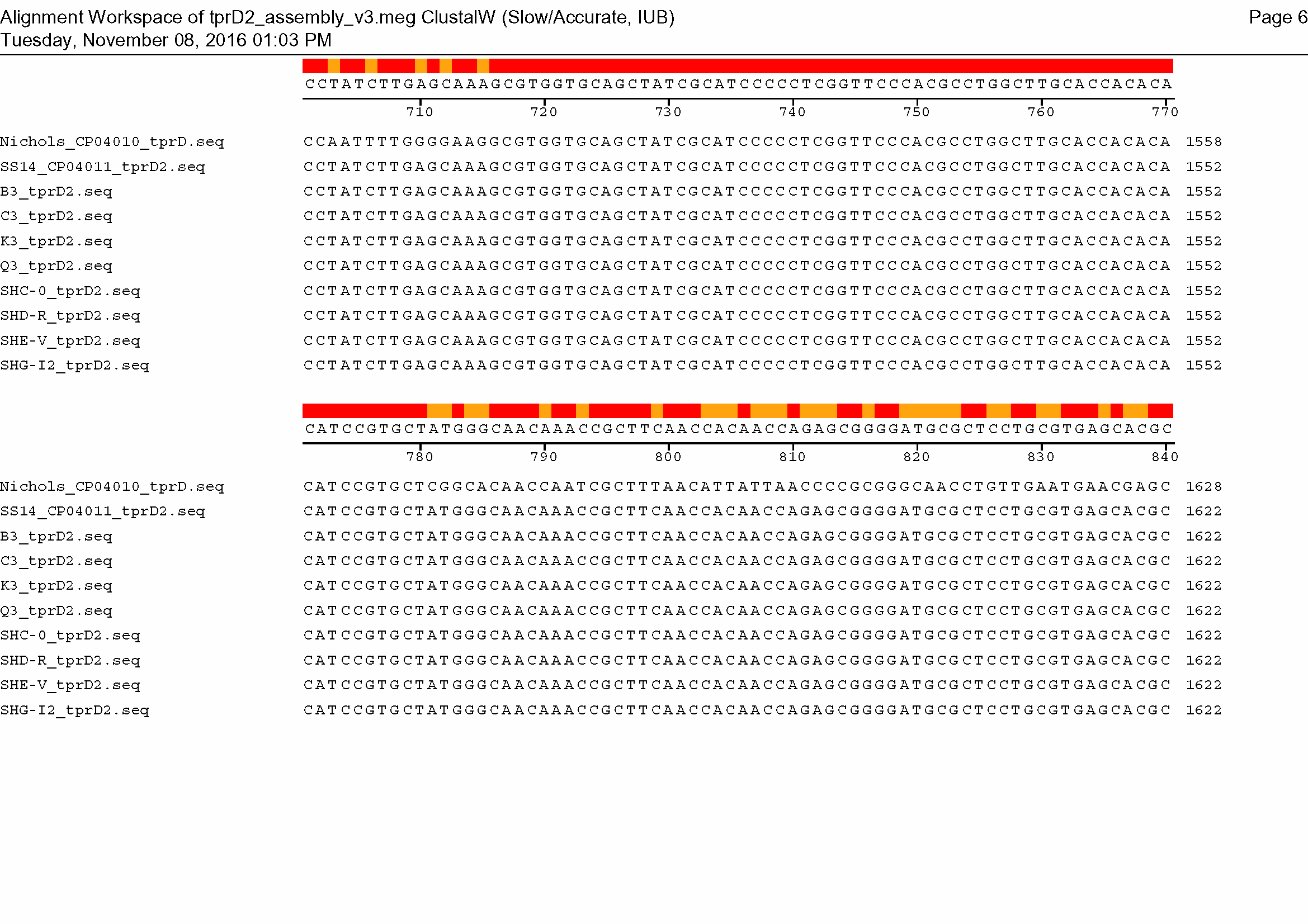


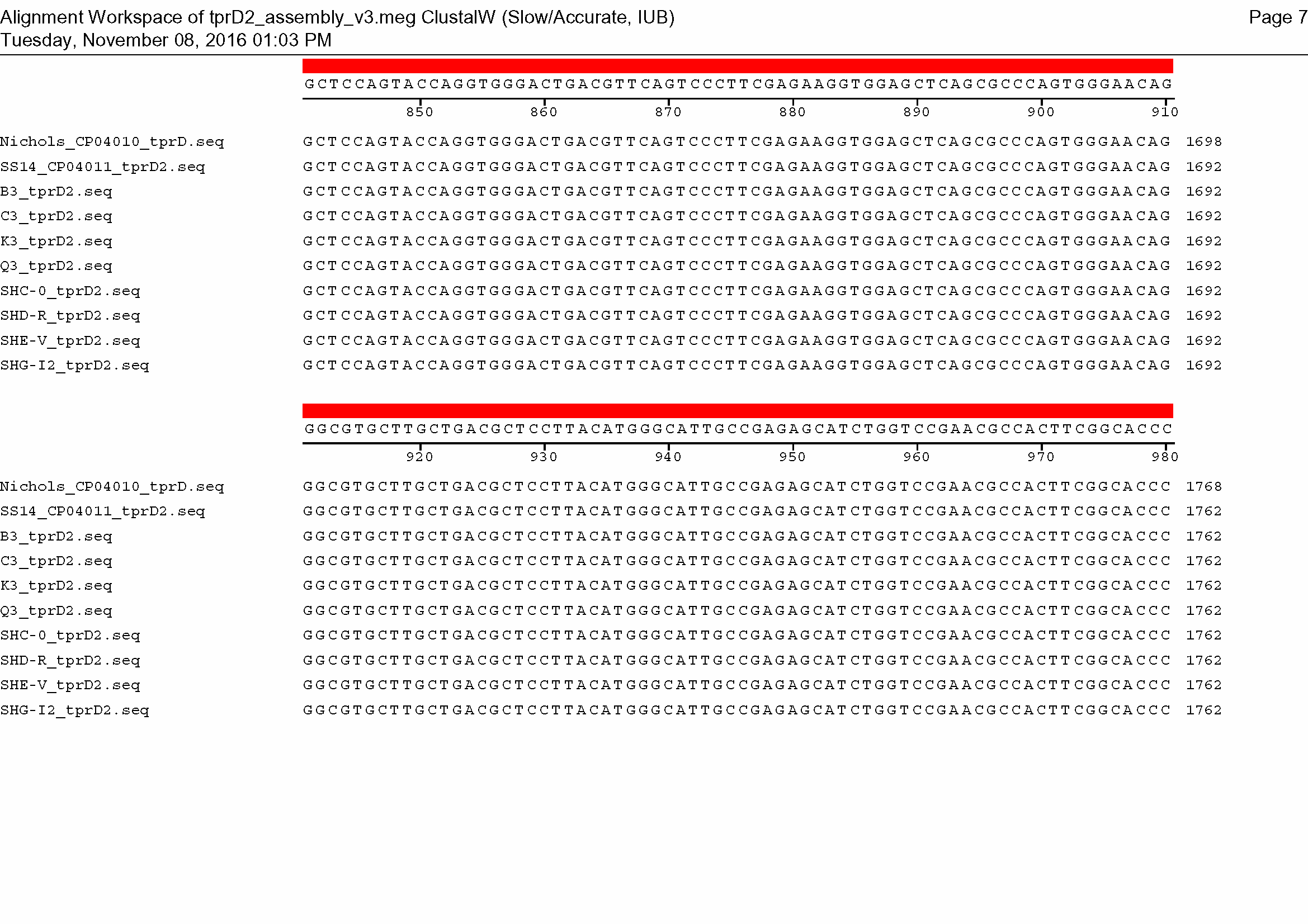


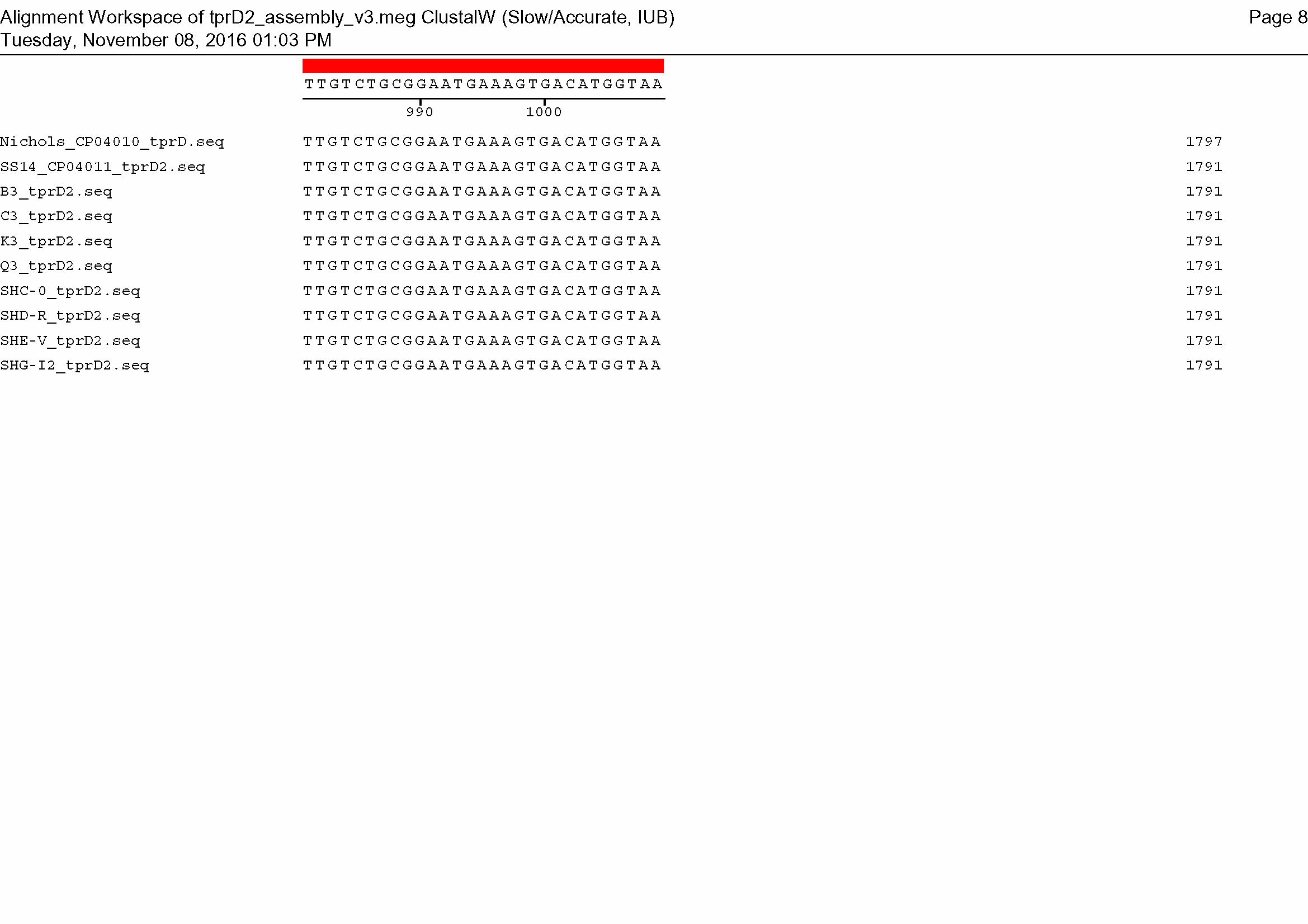

Supplement: Supplementary file 7 — Additional file 7. Alignment of tprD/tprD2 alleles. tprD and tprD2 alleles were downloaded from the NCBI GenBank database for each reference Nichols and SS14 TPA strain, CP004010.2 and CP004011.1 [6], respectively. While the Nichols reference genome harbors identical copies of tprC and tprD genes, the SS14 reference genome carries the tprD2 allele, which is not identical to the tprC gene and differs from the tprD allele by roughly 320 nucleotides. As shown in the alignment, we were able to identify the tprD2 allele (in positions 800–1791 according to the SS14 tprD2 allele) among the sequencing reads from the Chinese SRA data. The alignment was performed using SeqMan software (DNASTAR, Madison, WI, USA). [file 13104_2017_3106_MOESM7_ESM.doc]
