# Supplementary material for: Reanalysis of Chinese Treponema pallidum samples: all Chinese samples cluster with SS14-like group of syphilis-causing treponemes
Source: BMC Res Notes. 2018 Jan 11;11:16. doi: 10.1186/s13104-017-3106-7 (PMC5765698; doi:10.1186/s13104-017-3106-7)
Supplement: Supplementary file 8 — Additional file 8. The phylogenetic trees of the tp0136 and tp0548 genes. The phylogenetic trees were constructed using the Maximum Likelihood method based on the Tamura–Nei model. The bar scale represents the number of substitutions per site. The analysis involved 14 TPA nucleotide sequences including eight derived from the Chinese samples: SHC-0, SHD-R, SHE-V, SHG-I2, B3, C3, K3, and Q3. The T. pallidum subsp. pertenue Fribourg-Blanc sequence [1] was used as an outgroup. There were totals of 1547 and 1317 positions in the final dataset for tp0136 and tp0548 genes, respectively. For both genes, two separate clusters were identified: one cluster of Nichols-like TPA strains (TPA Lineage 1), and a second cluster of SS14-like TPA strains including all tested Chinese strains (TPA Lineage 3). Both clusters were supported by bootstrap values greater than 95%. [file 13104_2017_3106_MOESM8_ESM.doc]

**Additional file 8. The phylogenetic trees of the *tp0136* and *tp0548* genes.** The phylogenetic trees were constructed using the Maximum Likelihood method based on the Tamura-Nei model. The bar scale represents the number of substitutions per site. The analysis involved 14 TPA nucleotide sequences including eight derived from the Chinese samples: SHC-0, SHD-R, SHE-V, SHG-I2, B3, C3, K3, and Q3. The *T. pallidum* subsp. *pertenue* Fribourg-Blanc sequence [1] was used as an outgroup. There were totals of 1547 and 1317 positions in the final dataset for *tp0136* and *tp0548* genes, respectively. For both genes, two separate clusters were identified: one cluster of Nichols-like TPA strains (TPA Lineage 1), and a second cluster of SS14-like TPA strains including all tested Chinese strains (TPA Lineage 3). Both clusters were supported by bootstrap values greater than 95%.


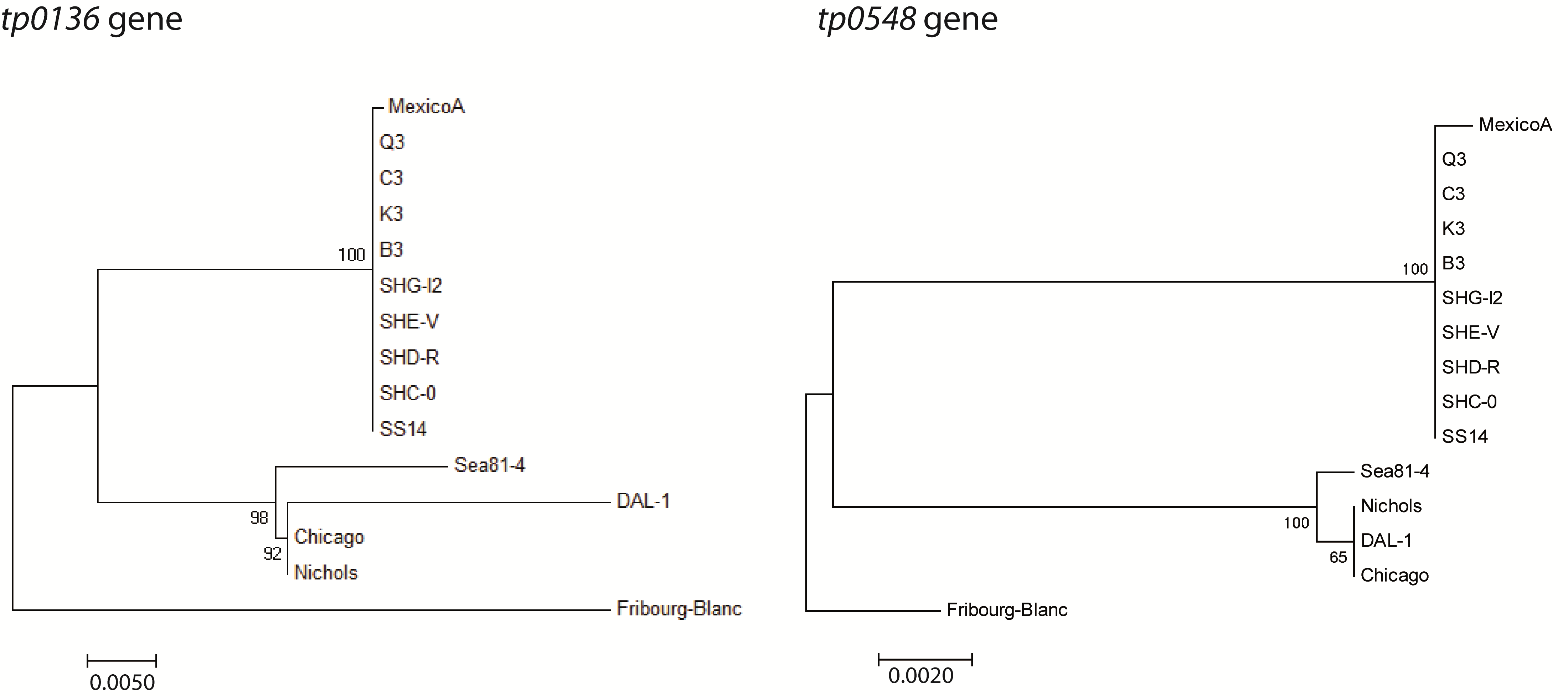


References

1. Zobaníková M, Strouhal M, Mikalová L, Čejková D, Ambrožová L, Pospíšilová P, et al. Whole genome sequence of the *Treponema* Fribourg-Blanc: unspecified simian isolate is highly similar to the yaws subspecies. PLoS Negl Trop Dis. 2013;7(4):e2172.
